# Dielectric Enhancement of Atomic Layer-Deposited Al_2_O_3_/ZrO_2_/Al_2_O_3_ MIM Capacitors by Microwave Annealing

**DOI:** 10.1186/s11671-019-2874-5

**Published:** 2019-02-11

**Authors:** Bao Zhu, Xiaohan Wu, Wen-Jun Liu, Shi-Jin Ding, David Wei Zhang, Zhongyong Fan

**Affiliations:** 1 Department of Materials Science, Fudan University, Shanghai, 200433 People’s Republic of China; 2 School of Microelectronics, Fudan University, Shanghai, 200433 People’s Republic of China

**Keywords:** Microwave annealing, Atomic layer deposition, Al_2_O_3_/ZrO_2_/Al_2_O_3_, MIM capacitors

## Abstract

For metal-insulator-metal (MIM) capacitors applicated in the fields of RF, DRAM, and analog/mixed-signal integrated circuits, a high capacitance density is imperative with the downscaling of the device feature size. In this work, the microwave annealing technique is investigated to enhance the dielectric characteristics of Al_2_O_3_/ZrO_2_/Al_2_O_3_ based MIM capacitors. The results show that the permittivity of ZrO_2_ is increased to 41.9 (~ 40% enhanced) with a microwave annealing at 1400 W for 5 min. The substrate temperature is lower than 400 °C, which is compatible with the back end of line process. The leakage current densities are 1.23 × 10^−8^ and 1.36 × 10^−8^ A/cm^2^ for as-deposited sample and 1400 W sample, respectively, indicating that the leakage property is not deteriorated. The conduction mechanism is confirmed as field-assisted tunneling.

## Background

Metal-insulator-metal (MIM) capacitors have been widely used in the fields of radio frequency (RF), dynamic random access memory (DRAM), and analog/mixed-signal integrated circuits. With the scaling down of the device feature size, it is desirable to obtain an ever higher capacitance density. For example, the capacitance density is required to be greater than 10 fF/μm^2^ according to the 2020 node of the International Technology Roadmap for Semiconductors (ITRS) [1]. As a consequence, a large number of high-κ materials have been investigated, such as HfO_2_ [2–6], ZrO_2_ [7–14], Ta_2_O_5_ [15–18], and TiO_2_ [19–24]. Among these high-κ materials, ZrO_2_ has a dielectric constant (κ) of 16~25 (monoclinic phase) and a bandgap of 5.8 eV. However, the κ value of ZrO_2_ can be enhanced to 36.8 and 46.6 when it is crystallized into cubic and tetragonal phase, respectively [25]. Hence, the capacitance density can be further increased. The microwave annealing (MWA) technique has been tremendously explored for the dopant activation in silicon [26–28] and the silicide formation [29, 30] due to its lower process temperature compared with conventional thermal processing techniques. In addition, Shih et al. [31] investigated the effect of MWA on electrical characteristics of TiN/Al/TiN/HfO_2_/Si MOS capacitors. Some key parameters such as equivalent oxide thickness, interface state density, and leakage current density were all improved.

In this work, the effect of MWA on electrical properties of TaN/Al_2_O_3_/ZrO_2_/Al_2_O_3_/TaN (TaN/A/Z/A/TaN) MIM capacitors is investigated. With the usage of MWA, the permittivity of ZrO_2_ is remarkably enhanced and the leakage current density is slightly increased. Moreover, the underlying conduction mechanism is also studied.

## Methods

Firstly, a 500-nm-thick SiO_2_ film was grown onto Si substrate by PECVD, followed by deposition of TaN (20 nm)/Ta (100 nm) films, and TaN was grown by sputtering Ta target in N_2_/Ar plasma. Subsequently, the Si wafer coated with the TaN/Ta films was transferred into the ALD chamber, and the nano-stack of Al_2_O_3_ (2 nm)/ZrO_2_ (20 nm)/Al_2_O_3_ (2 nm) were deposited at 250 °C. Al_2_O_3_ and ZrO_2_ films were grown from Al (CH_3_)_3_/H_2_O and [(CH_3_)_2_N]_4_Zr/H_2_O, respectively. It is worth mentioning that an ultrathin Al_2_O_3_ layer between the bottom TaN electrode and the ZrO_2_ layer was inserted to restrain the formation of interfacial layer during ALD and post-deposition annealing. Afterwards, the samples were subject to the microwave annealing. MWA was performed in a DSGI octagonal chamber at 5.8 GHz. During annealing, the samples were placed at the middle of the chamber, where the electromagnetic field is most uniform. The in situ temperature of the samples was monitored by a Raytek XR series infrared pyrometer facing the backside of the samples. The power was varied from 700 W to 1400 W with a fixed annealing time of 5 min. Finally, a 100-nm-thick TaN top electrode was formed in turn by reactive sputter, lithography, and reactive ion etching.

The ALD film thicknesses were measured with an ellipsometer (SOPRA GES 5E) and confirmed by transmission electron microscope (TEM). Capacitance-voltage (*C-V*) was measured by a precision impedance analyzer (Agilent 4294A) with a 50 mV AC amplitude. Current-voltage (*I-V*) measurements were performed with a semiconductor device analyzer (Agilent B1500) in a dark box. The bias was applied to the top electrode.

## Results and Discussion

**Fig. 1:**
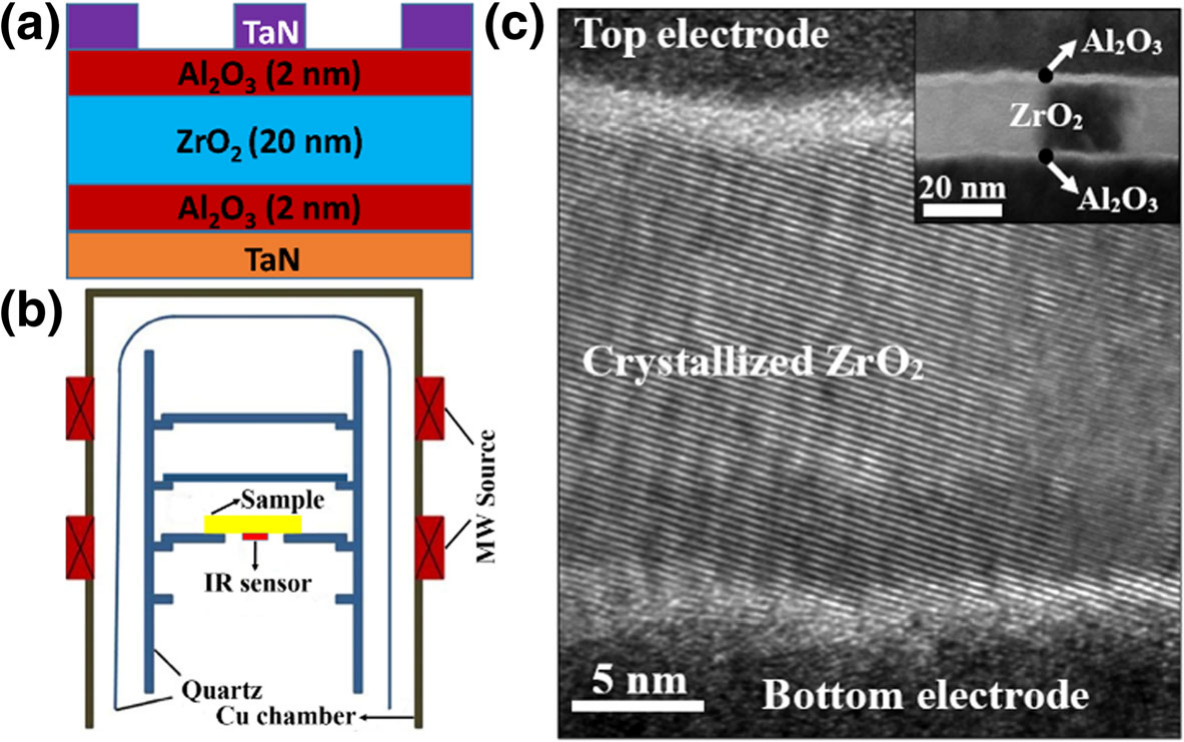
**a** The schematic structure of Al_2_O_3_/ZrO_2_/Al_2_O_3_-based MIM capacitor. **b** The schematic structure of the MWA chamber. **c** TEM picture of Al_2_O_3_/ZrO_2_/Al_2_O_3_-based MIM capacitor with MWA at 1400 W for 5 min

**Fig. 2:**
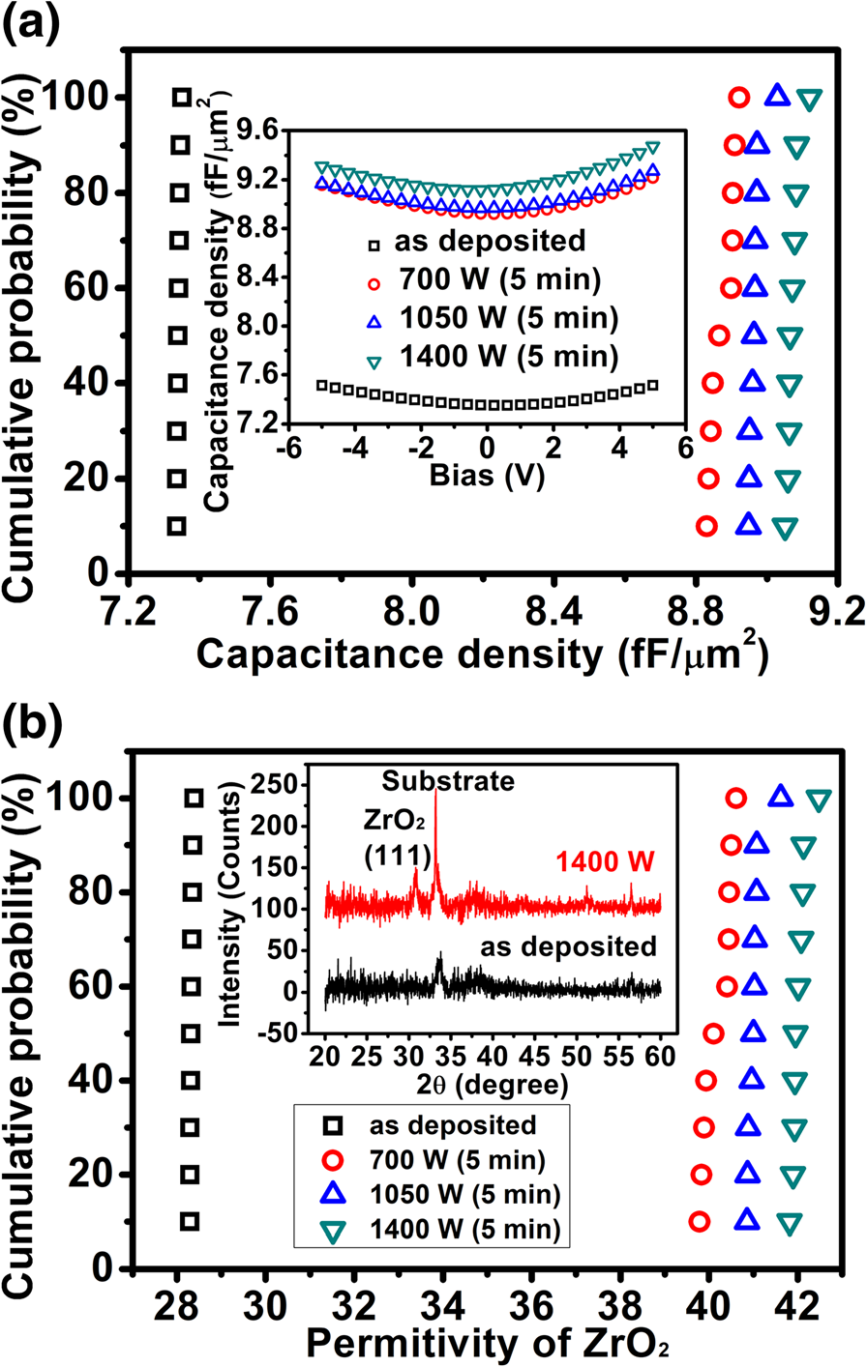
**a** The cumulative probability plot of the capacitance density for different samples; the inset displays the capacitance density against the bias. **b** The cumulative probability plot of the permittivity of ZrO_2_ for different samples; the inset exhibits the XRD patterns of the as-deposited and 1400 W samples

The schematic structures of the A/Z/A based MIM capacitor and the MWA chamber are shown in Fig. 1a and b, respectively. Figure 1c exhibits the cross-sectional TEM image of the A/Z/A-based MIM capacitor which is subject to the MWA at 1400 W for 5 min. It is observed that the ZrO_2_ layer is fully crystallized and the stacked layers can be distinguished clearly, see the inset. Figure 2a shows the cumulative probability plot of the capacitance density at different annealing power. The results show that the capacitance densities of the MIM capacitors are 7.34, 8.87, 8.96, and 9.06 fF/μm^2^ respectively for 0, 700, 1050, and 1400 W at a 50% cumulative probability. Therefore, the capacitance density is increased under the effect of microwaves. The very narrow distribution of the capacitance density for the A/Z/A stack MIM capacitors with MWA indicates very good annealing uniformity. The inset in Fig. 2a exhibits the typical CV curves of all the samples. Excluding the effect of Al_2_O_3_ (κ ≈ 8), the dielectric constants of the ZrO_2_ films are extracted as 28.3, 40.1, 41, and 41.9 for 0, 700, 1050, and 1400 W, respectively, revealed by Fig. 2b. Regarding the microwave power of 1400 W, the dielectric constant of the ZrO_2_ film increases by 40% compared with the as-deposited sample. The significant enhancement of the permittivity of ZrO_2_ can be ascribed to the high-degree crystallization during the microwave annealing, shown in Fig. 1c. As mentioned above, the dielectric constant of ZrO_2_ can be enhanced to 36.8 and 46.6 when it is crystallized into cubic and tetragonal phase, respectively [25]. Hence, the XRD measurement was performed to further investigate the mechanism of the dielectric constant enhancement. As exhibited in the inset of Fig. 2b, a peak existed at ~ 30. 7° after the MWA processing at 1400 W, indicating the appearance of the tetragonal phase (111) in ZrO_2_ [32, 33]. The presence of this tetragonal phase is responsible for the enhancement of the dielectric constant from 28.3 to over 40.

**Fig. 3:**
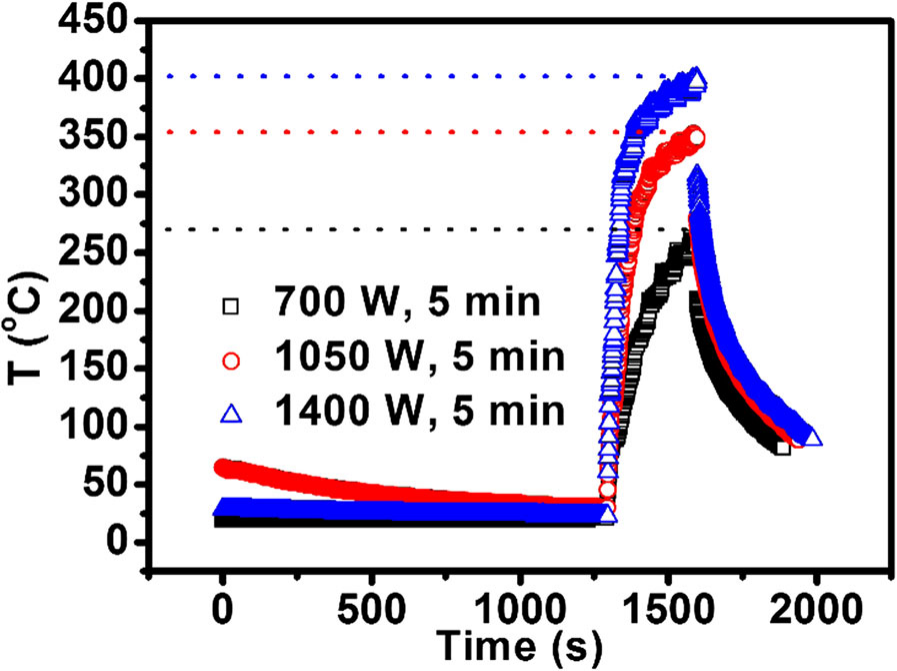
The curves of the substrate temperature for different samples during the microwave annealing

Since the MIM capacitors are fabricated in the back end of line (BEOL) of integrated circuits, the process temperature must be lower than 400 °C [34]. As shown in Fig. 3, the temperature curves of MWA indicate that the highest temperatures of the substrate are 260, 350, and 400 °C for 700, 1050, and 1400 W, respectively. Therefore, MWA is compatible with the CMOS process from the viewpoint of process temperature. Furthermore, in the previous work [13], Al_2_O_3_ (2 nm)/ZrO_2_ (20 nm)-based MIM capacitors were subject to rapid thermal annealing (RTA) at 420 °C for 10 min in N_2_/H_2_ ambient and the resulting dielectric constant of ZrO_2_ was evaluated as 40. For RTA, the annealing time was kept constant at 420 °C for 10 min, so the thermal budget was much larger compared with MWA. For MWA [35, 36], dipole polarization is thought to be the most important mechanism for energy transfer at the molecular level. When materials in contact have different dielectric properties, microwaves will selectively couple with the higher dielectric loss materials. In contrast, conventional RTA transfers heat most efficiently to materials with high conductivity.

**Fig. 4:**
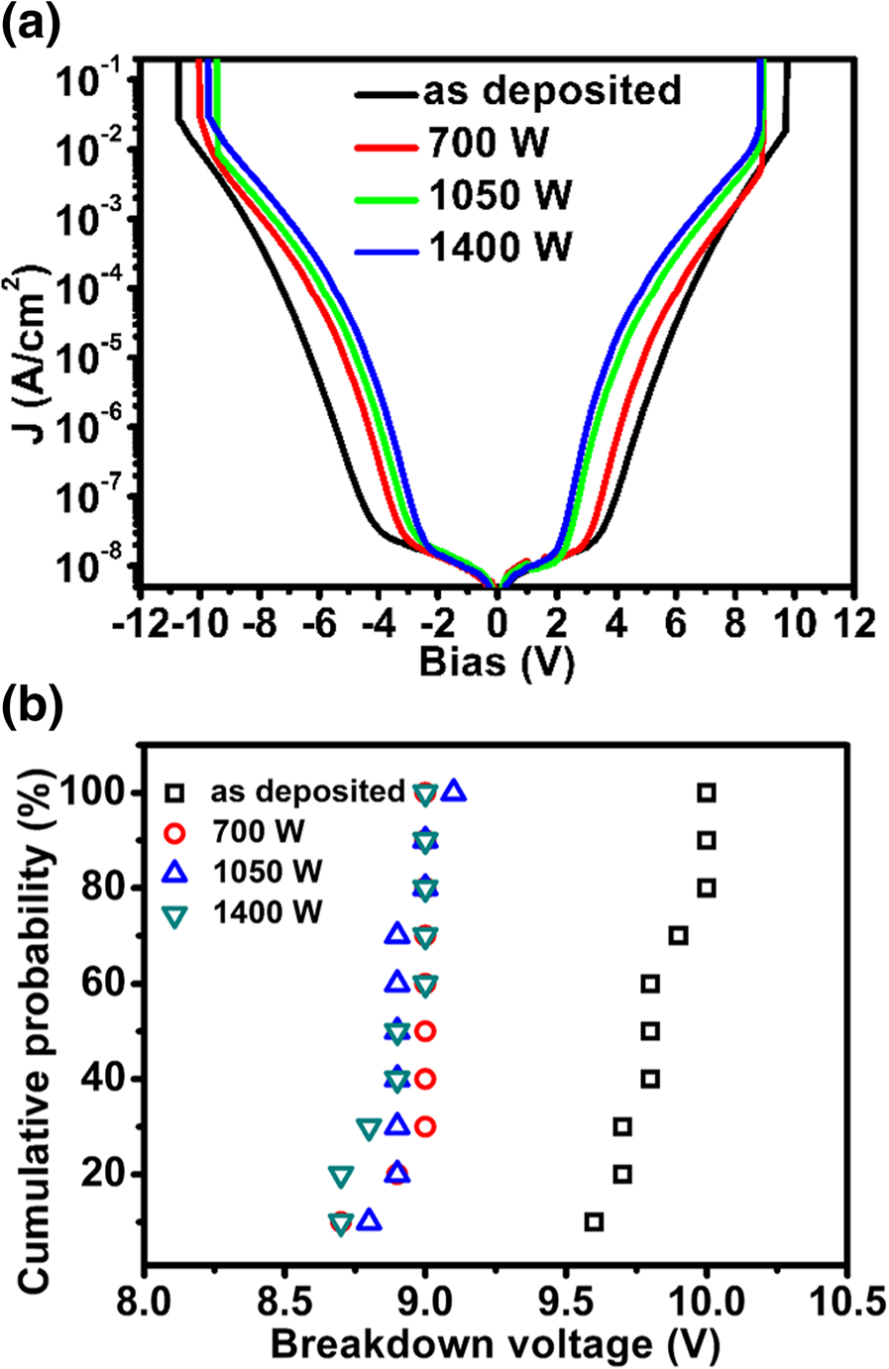
**a** The plot of the leakage current density (*J*) vs bias and **b** the cumulative probability plot of the breakdown voltage for different samples

Leakage current is another important parameter for MIM capacitors. As shown by Fig. 4a, the leakage current curve can be divided into two sections for all the samples since there is an obvious turning point, indicating different electron conduction mechanisms. As for the samples with MWA processing, the voltage corresponding to the turning point is smaller compared with the as-deposited sample. Table 1 lists the leakage current density at ± 4 V for all the samples. Take 4 V for example, the leakage current density is increased from 1.06 × 10^−7^ to 1.92 × 10^−5^ A/cm^2^, i.e., two orders of amplitude enhanced when the microwave power is augmented from 0 to 1400 W. Due to a high crystallization of the ZrO_2_ film, a large number of grain boundaries will appear and serve as the leaky path, thus enhancing the electron conduction under a high electric field. However, considering a working voltage of 2 V, the leakage current densities are 1.23 × 10^−8^ and 1.36 × 10^−8^ A/cm^2^ for as-deposited sample and 1400 W sample, respectively. Obviously, the microwave annealing has little effect on the leakage performance under a low electric field. Furthermore, the breakdown voltage was extracted from the *I*-*V* test and plotted in Fig. 4b. For the as-deposited sample, the breakdown voltage is about 9.8 V at a 50% cumulative probability. With the application of MWA, the breakdown voltage is reduced to ~ 9 V. This reduction of breakdown voltage could be related to the change of the ZrO_2_ microstructure.

**Table 1:** The leakage current density (*J*) at ± 4 V for all the samples

	As-deposited	700 W	1050 W	1400 W
*J*@4 V (A/cm^2^)	1.06 × 10^−7^	6.68 × 10^−7^	7.63 × 10^−6^	1.92 × 10^−5^
*J*@-4 V (A/cm^2^)	3.41 × 10^−8^	3.30 × 10^−7^	1.20 × 10^−6^	3.48 × 10^−6^

In order to further understand the effect of MWA on the leakage current, the conduction mechanisms of the MIM capacitors are investigated. Based on the previous research on Al_2_O_3_ (2 nm)/ZrO_2_ (20 nm)-based MIM capacitor [13, 14], the dominant conduction mechanism in a high electric field was confirmed as field-assisted tunneling (FAT). For FAT which is trap-related tunneling, electrons are captured by the traps in the insulator firstly and then tunnel to the conduction band of the insulator directly [37]. In the current work, the Al_2_O_3_ and ZrO_2_ films in the A/Z/A-based MIM capacitors were deposited by the same conditions, so the leakage current is probably predominant by FAT as well. The FAT model can be expressed by Eq. (1) [37]

1 $$ J={AE}^2\exp \left(-\frac{8\pi \sqrt{2{m}^{\ast }q{\varphi}_t^3}}{3 hE}\right) $$

where *A* is a constant, *E* is the electric field, *q* is the electronic charge, m* represents the effective electron mass (about 0.25 *m*_0_, where *m*_0_ is the free electron mass), *k* is the Boltzmann constant, *φ*_*t*_ is the energy barrier separating traps from the conduction band, and *h* is the Planck’s constant.

**Fig. 5:**
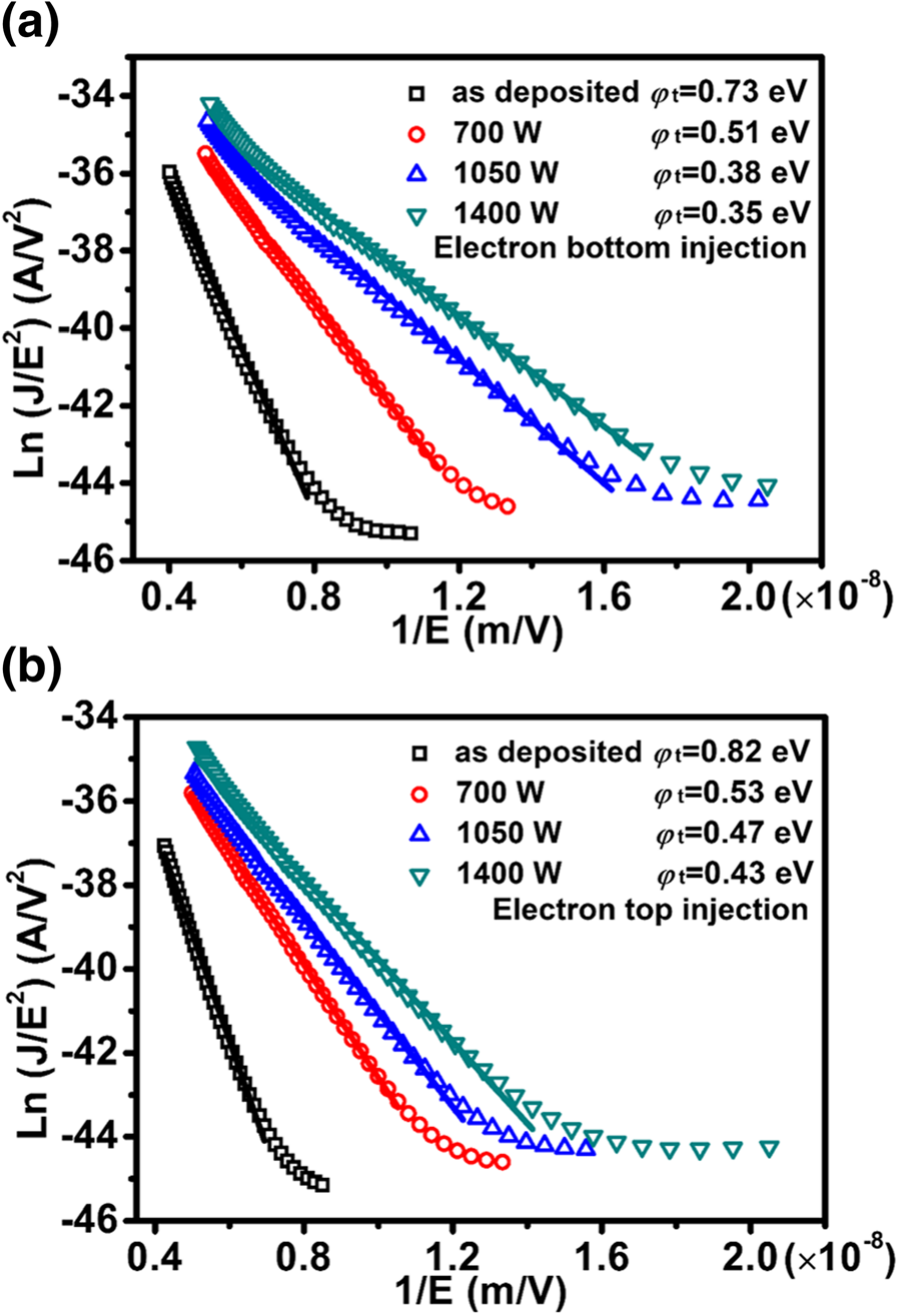
The plot of Ln (*J*/*E*^2^) vs 1/*E* for different samples. **a** Electron bottom-injection and **b** electron top-injection

In terms of the stacked dielectrics, the electric field applied to each layer differs from each other because of different permittivity and thickness. Hence, using the average electric field across the entire stack will bring about severe errors while discussing the conduction mechanism. As a consequence, the electric field across the ZrO_2_ layer must be extracted accurately. The electric fields across ZrO_2_ are 3.125 × 10^7^ × *V*_stack_, 2.5 × 10^7^ × *V*_stack_, 2.47 × 10^7^ × *V*_stack_, and 2.44 × 10^7^ × *V*_stack_ respectively for as-deposited, 700 W, 1050 W, and 1400 W sample according to the Gauss law and Kirchhoff voltage law [38, 39]:

2 $$ \left\{\begin{array}{c}{k}_A{E}_A={\kappa}_Z{E}_Z\\ {}{d}_A{E}_A+{d}_Z{E}_Z={V}_{stack}\end{array}\right. $$

where *k*_A_ and *κ*_Z_ represent the dielectric constants of Al_2_O_3_ and ZrO_2_, respectively; *E*_A_ and *E*_Z_ denote the electric fields across Al_2_O_3_ and ZrO_2_, respectively; *d*_A_ and *d*_Z_ equal the thicknesses of Al_2_O_3_ and ZrO_2_, respectively; and *V*_stack_ is the voltage applied to the stack. Accordingly, Ln (*J*/*E*_Z_^2^) versus 1/*E*_Z_ was arbitrarily plotted in Fig. 5, where a straight line fitting was achieved in the high field region for each sample under electron bottom-injection (see Fig. 5a) or electron top-injection (see Fig. 5b). This means that the FAT mechanism is dominated at high electric fields. The extracted *φ*_t_ is 0.73, 0.51, 0.38, and 0.35 eV respectively for as-deposited, 700 W, 1050 W, and 1400 W sample under electron bottom-injection. In terms of electron top-injection, the corresponding *φ*_t_ is 0.82, 0.53, 0.47, and 0.43 eV, respectively. Therefore, some shallow traps are induced by MWA. The shallow traps are reported to arise from the grain boundary defects that can introduce additional electronic states near the conduction band [40]. In addition, the conduction mechanism at low fields is most likely trap-assisted tunneling (TAT).

## Conclusions

Atomic layer-deposited Al_2_O_3_/ZrO_2_/Al_2_O_3_ nano-stack is used as the insulator of the MIM capacitors. With the effect of MWA at 1400 W for 5 min, the capacitance density is increased to 9.06 fF/μm^2^, approximately 23.4% of capacitance enhanced. Decoupling the influence of Al_2_O_3_, the dielectric constant is deduced as 41.9 for 1400 W sample (~ 40% of permittivity increased). Such enhancement of the permittivity is originated from a high crystallization of the ZrO_2_ film. In addition, the substrate temperature is lower than 400 °C, which enables MWA compatible with the BEOL process. This lower substrate temperature can be attributed to the selective heating on the materials of MWA. In terms of a working voltage of 2 V, the leakage current densities are 1.23 × 10^−8^ and 1.36 × 10^−8^ A/cm^2^ for as-deposited sample and 1400 W sample, respectively. The dominated conduction mechanism in the high electric fields is confirmed as a FAT process. The leakage current in the low electric fields is likely dictated by TAT. Based on the above facts, the microwave annealing is a promising technique used in the CMOS process to enhance the dielectric performance of the MIM capacitors.

## Abbreviations

A/Z/A: Al_2_O_3_/ZrO_2_/Al_2_O_3_
ALD: Atomic layer deposition
BEOL: Back end of line
*C-V*: Capacitance-voltage
DRAM: Dynamic random access memory
FAT: Field-assisted tunneling
ITRS: International Technology Roadmap for Semiconductors
*I-V*: Current-voltage
MIM: Metal-insulator-metal
MWA: Microwave annealing
PECVD: Plasma enhanced chemical vapor deposition
RF: Radio frequency
RTA: Rapid thermal annealing
TAT: Trap-assisted tunneling
TEM: Transmission electron microscope
